# Diverse Medical School Class and Learner Satisfaction

**DOI:** 10.1001/jamanetworkopen.2025.58240

**Published:** 2026-03-10

**Authors:** Mytien Nguyen, Sarwat I. Chaudhry, Gbenga Ogedegbe, David Henderson, Dowin Boatright

**Affiliations:** 1 Department of Immunobiology, Yale University School of Medicine, New Haven, Connecticut; 2 Section of General Internal Medicine, Department of Medicine, Yale School of Medicine, New Haven, Connecticut; 3 Institute for Excellence in Health Equity, New York University Grossman School of Medicine, New York; 4 Department of Family Medicine, University of Connecticut School of Medicine, Hartford; 5 Department of Emergency Medicine, New York University Langone Health, New York

## Abstract

This cross-sectional study examines the association between medical school students’ diversity and graduates’ reported satisfaction with their educational experience using data from the Association of American Medical Colleges.

## Introduction

Racially and ethnically diverse medical school student bodies may improve students’ cultural competence, reduce bias, and enhance readiness to serve diverse patients, including White students.^1^ However, few studies have examined the association between student body diversity and learner satisfaction. This study examines the association between student diversity—as measured by underrepresented in medicine (URiM; eg, Alaska Native, American Indian, Black, Hispanic, Native Hawaiian, and Pacific Islander) representation—with graduates’ report of high satisfaction with their educational experience.

## Methods

Graduation Questionnaire (GQ) responses for medical school matriculants from 2013 to 2014 to 2023 to 2024 were retrieved from the Association of American Medical Colleges. Students self-reported their race and ethnicity to the American Medical College Application Service.

In the GQ, students are asked to indicate agreement (strongly agree to strongly disagree) with the following statement: “Overall, I am satisfied with the quality of my medical education.” High satisfaction was defined as those reporting strongly agree. We define medical school racial and ethnic diversity as the proportion of URiM students by medical school during the study, categorized into terciles from lowest diversity (1st tercile) to highest diversity (3rd tercile).

We estimated adjusted relative risks (aRRs) by performing multivariable population-averaged Poisson regression with a log link and robust standard errors using generalized estimating equations to account for within-school clustering of students, and adjusted for school ownership and geographical region. Historically Black Colleges and Universities and Puerto Rican medical schools were excluded due to their missions to train diverse physicians. Sensitivity analysis was conducted examining class-level URiM representation, defined as diversity of students’ matriculating class instead of the entire student body. This cross-sectional study was exempt from review and informed consent per criteria by the New York University Langone Health institutional review board and follows the STROBE reporting guideline. Statistical significance was set at *P* <.05, and all tests were 2-sided. Data were analyzed from May to September 2025 using Stata version 19.5 (StataCorp).

## Results

Among 114 168 students, 22 834 (20.0%) identify as Asian, 12 018 (10.5%) as URiM, and 62 865 (55.1%) as White. Among these students, 7523 Asian (32.9%), 4460 URiM (37.1%), and 27 084 White (43.1%) students reported strongly agree with satisfaction with their medical education. For all students, strong agreement with satisfaction in the quality of medical education was higher at the second and third URiM terciles (first, 14 569 [37.8%]; second, 16 761 [40.9%]; third, 14 136 [40.6%]; *P* < .001). This difference was seen for all racial and ethnic groups (first, second, and third tercile: Asian students, 1979 [30.3%], 2829 [33.9%], 2715 [34.0%]; URiM students, 715 [32.0%], 1572 [37.9%], 2173 [38.4%]; White students, 10 075 [40.6%], 9960 [44.4%], 7049 [45.0%]; *P* < .001) (Figure). Sensitivity analysis examining class-level URiM representation showed similar trends.

**Figure.**
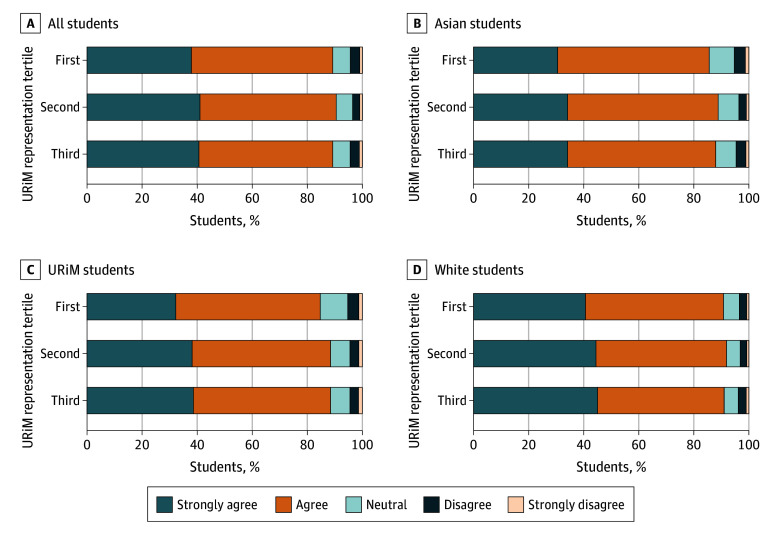
Reported High Satisfaction With Medical Education by School-Level URiM Representation Rates of reported satisfaction with medical education by racial and ethnic groups, and for all students in aggregate. URiM terciles: first (2.400%-9.999%), second (10.000%-14.106%), third (14.107%-46.600%). URiM indicates underrepresented in medicine (eg, Alaska Native, American Indian, Black, Hispanic, Native Hawaiian, and Pacific Islander individuals).

In the adjusted model, compared with their counterparts in the lowest URiM tercile schools, the relative risk of strong agreement with satisfaction in the quality of their medical education was significantly higher among URiM and White students in the second and third URiM tercile for representational diversity (URiM: second tercile aRR, 1.18 [95% CI, 1.02-1.36]; *P* = .02; third tercile aRR, 1.15 [95% CI, 1.001-1.33]; *P* = .048; White: second tercile aRR, 1.19 [95% CI, 1.05-1.35]; *P* = .004; third tercile aRR, 1.14 [95% CI, 1.01-1.29]; *P* = .047). No difference was found among Asian students (Table).

**Table.** Adjusted Relative Risk of High Satisfaction With Medical Education by School Characteristics

Characteristics	Adjusted relative risk (95% CI)			
	All students	Asian	URiM	White
% URiM				
First (2.400-9.999)	1 [Reference]	1 [Reference]	1 [Reference]	1 [Reference]
Second (10.000-14.106)	1.15 (1.02-1.31)^a^	1.17 (0.93-1.48)	1.18 (1.02-1.36)^a^	1.19 (1.05-1.35)^a^
Third (14.107-46.600)	1.11 (0.97-1.26)	1.18 (0.91-1.53)	1.15 (1.001-1.33)^a^	1.14 (1.01-1.29)^a^
School ownership				
Public	1 [Reference]	1 [Reference]	1 [Reference]	1 [Reference]
Private	1.05 (0.90-1.22)	0.95 (0.74-1.23)	0.86 (0.75-0.98)	1.00 (0.88-1.13)
School region				
Central	1 [Reference]	1 [Reference]	1 [Reference]	1 [Reference]
Northeast	1.05 (0.89-1.23)	1.09 (0.81-1.47)	0.99 (0.82-1.19)	1.04 (0.89-1.21)
Southern	1.02 (0.88-1.19)	0.98 (0.74-1.30)	1.03 (0.87-1.22)	1.05 (0.91-1.21)
Western	1.01 (0.84-1.21)	1.10 (0.77-1.56)	1.15 (0.95-1.39)	1.00 (0.84-1.20)

Abbreviation: URiM, underrepresented in medicine (eg, Alaska Native, American Indian, Black, Hispanic, Native Hawaiian, and Pacific Islander).

^a^ *P* < .05.

## Discussion

We observed a positive association between URiM representation and student reports of high satisfaction with medical education for White and URiM students. The positive association between URiM representation and high satisfaction with medical education among White students is noteworthy and challenges assertions that diversity disadvantages White students to the benefit of URiM students. Prior research demonstrates that racially diverse learning environments confer measurable benefits for White students, including stronger critical thinking skills, improved academic engagement, and greater preparedness for working in diverse settings.^1,2,3,4^

This study has limitations. These findings describe associations and cannot establish causation, and unmeasured confounding may explain some of the observed associations. Satisfaction was self-reported and may be influenced by unmeasured personal or contextual factors. URiM representation was assessed at the institutional level and may not reflect students’ individual experiences. Furthermore, in our adjusted model, we found no association between URiM representation and report of high satisfaction among Asian students. URiM representation may not capture dimensions of inclusion salient to Asian students. Additionally, Asian students represent a heterogeneous group, underscoring the importance of examining satisfaction among disaggregated Asian subgroups.^5^

Recent literature demonstrates that most medical school matriculants place value on the representational diversity of their peers when selecting a medical school.^6^ Findings from our study could be used by medical school leadership to guide evidence-based interventions to optimize the medical school learning environment and enhance student satisfaction with training.

## Notes

Supplement.
Data Sharing Statement
