# Supplementary material for: Diverse Medical School Class and Learner Satisfaction
Source: JAMA Netw Open. 2026 Mar 10;9(3):e2558240. doi: 10.1001/jamanetworkopen.2025.58240 (PMC12976789; doi:10.1001/jamanetworkopen.2025.58240)
Supplement: Supplement. — Data Sharing Statement [file jamanetwopen-e2558240-s001.pdf]

Data Sharing Statement

Nguyen. Diverse Medical School Class and Learner Satisfaction. *JAMA Netw Open*. Published March 10, 2026. doi:10.1001/jamanetworkopen.2025.58240

Data
Data available: No
